# Myocardial bridging—an unusual cause of Wellens syndrome: A case report

**DOI:** 10.1097/MD.0000000000022491

**Published:** 2020-10-09

**Authors:** Anamaria Avram, Valentin Chioncel, Suzana Guberna, Irina Cuciureanu, Radu Constantin Brezeanu, Catalina Liliana Andrei, Crina Sinescu

**Affiliations:** a Carol Davila University of Medicine and Pharmacy; b Bagdasar-Arseni Emergency Clinical Hospital, Bucharest, Romania.

**Keywords:** angina pectoris, biphasic T waves, myocardial bridge, Wellens syndrome

## Abstract

**Rationale:**

Coronary chest pain is usually ischemic in etiology and has various electrocardiographic presentations. Lately, it has been recognized that myocardial bridging (MB) with severe externally mechanical compression of an epicardial coronary artery during systole may result in myocardial ischemia. Such a phenomenon can be associated with chronic angina pectoris, acute coronary syndromes (ACS), coronary spasm, ventricular septal rupture, arrhythmias, exercise-induced atrioventricular conduction blocks, transient ventricular dysfunction, and sudden death.

**Patient concerns:**

We report the case of a 58-year-old woman presenting with recurrent episodes of constrictive chest pain during exercise within the last 2 weeks. Except for obesity, general and cardiovascular clinical examination on admission were normal.

**Diagnoses:**

The resting 12 lead electrocardiogram (ECG) revealed changes typically for Wellens syndrome. High-sensitive cardiac troponin I was normal. We established the diagnosis of low-risk non-ST-segment elevation acute coronary syndrome with a Global Registry of Acute Coronary Events risk score of 92 points.

**Interventions:**

The patient underwent coronary angiography, who showed subocclusive dynamic obstruction of the left anterior descending artery due to MB.

**Outcomes:**

The patient was managed conservatively. Her hospital course was uneventful and she was discharged on pharmacological therapy (clopidogrel, bisoprolol, amlodipine, atorvastatin, and metformin) with well-controlled symptoms on followup.

**Lessons:**

MB is an unusual cause of myocardial ischemia. Wellens syndrome is an unusual presentation of ACS. We present herein a rare case of Wellens syndrome caused by MB. This case highlights the importance of subtle and frequently overseen ECG findings when assessing patients with chest pain and second, the importance of considering nonatherosclerotic causes for ACS.

## 1 Introduction

Myocardial bridging (MB) is an unusual cause of myocardial ischemia. It refers to a fascicle of myocardial tissue that overrides a segment of a coronary artery which thus becomes “tunneled” within the myocardium and exposed to extrinsec mechanical compression during systole. MB has been considered for a long time a benign congenital anomaly, but recently it has been recognized that it can be associated with angina pectoris (AP), acute coronary syndromes (ACS), coronary spasm, ventricular septal rupture, arrhythmias (including supraventricular and ventricular tachycardia), exercise-induced atrioventricular conduction blocks, transient ventricular dysfunction, and sudden death.^[1]^

Wellens syndrome is an unusual presentation of ACS. It has been initially described in the early 1980s by de Zwaan et al^[2]^ and encompasses a series of electrocardiogram (ECG) findings such as biphasic or deep negative T waves. These ECG changes usually appear in the anterior leads in patients with chest pain in whom coronary angiography reveals critical stenosis of the left anterior descending (LAD), often in the proximal segment.

We report the case of a 58-year-old woman with an unusual presentation of ACS—Wellens syndrome—due to an unusual cause—MB.

## 2 Case presentation

### 2.1 Patient information

A 58-year-old obese woman presented to the emergency department of our hospital with recurrent episodes of constrictive chest pain during exercise within the last 2 weeks (de novo AP). She described similar symptomatology a few years ago, but she did not have any medical evaluation at that time. The patient denied shortness of breath during daily activities, dizziness, or syncope. Her past medical history revealed type 2 diabetes mellitus, arterial hypertension, and dyslipidemia, with an estimated 10-year risk of fatal cardiovascular disease of 3% according to SCORE risk chart. She was on chronic ambulatory medication with bisoprolol 5 mg quaque die (QD), perindopril 5 mg QD, atorvastatin 20 mg QD, and metformin 1000 mg bis in die with good medication adherence. There was no family history of cardiac diseases.

### 2.2 Clinical findings

Focused clinical examination on admission revealed class I obesity (height = 156 cm, weight = 80 kg, body mass index  = 33 kg/m^2^); blood pressure was 120/65 mm Hg, heart rate was 56 bpm, heart and lung sounds were normal with no audible cardiac murmurs, respiratory rate was 17 breaths/min and peripheral oxygen saturation was 98% in ambiental air.

### 2.3 Diagnostic assessment

Laboratory workup revealed impaired fasting glucose (112 mg/dL = 6.2 mmol/L), high values for total cholesterol (210 mg/dL = 5.43 mmol/L), LDL-cholesterol (157 mg/dL = 4 mmol/L) and tryglicerides (157 mg/dL = 1.76 mmol/L), high NTproBNP (1120 pg/mL) and normal myocardial necrosis markers, including serial determination of myoglobin (33–28 ng/mL, cut-off value 60 ng/mL), high-sensitive cardiac troponin I (23-8-13 ng/L, cut-off value 54 ng/L), and creatinkinase (CK/CK-MB = 61/21– 84/15–52/17 UI/L, cut-off value 280/22 U/l). Routine liver and kidney function blood tests were within normal range (serum creatinine: 0.85 mg/dL = 75 μmol/L, blood urea nitrogen: 15.85 mg/dL = 5.66 mmol/L, AST = 32 U/L, ALT = 47 U/L).

**Figure 1:**
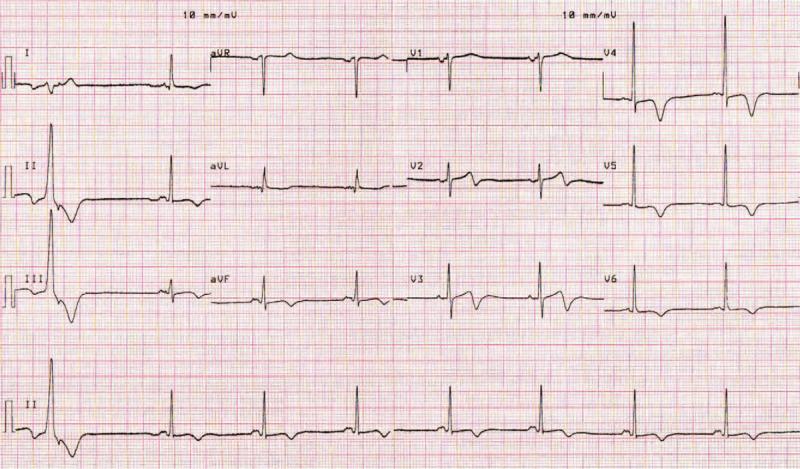
ECG on admission showing type A Wellens pattern. ECG = electrocardiogram.

The resting ECG on admission during pain-free interval showed biphasic T-waves with terminal negative component (+/−) in leads V2 to V6, DIII, aVF and minimal ST depression in leads DI and aVL (Fig. 1).

Transthoracic echocardiography revealed interventricular septum and anterior wall hypokinesia with preserved global left ventricular ejection fraction (55%) and mild mitral regurgitation.

We established the diagnosis of low-risk non-ST-segment elevation acute coronary syndrome with a Global Registry of Acute Coronary Events risk score of 92 points.

### 2.4 Therapeutic intervention

**Figure 2:**
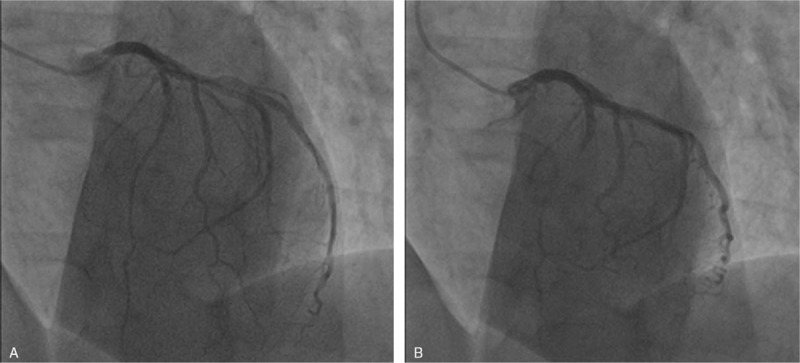
Coronary angiography, incidence left anterior oblique (LAO) during diastole (A) and systole (B).

**Figure 3:**
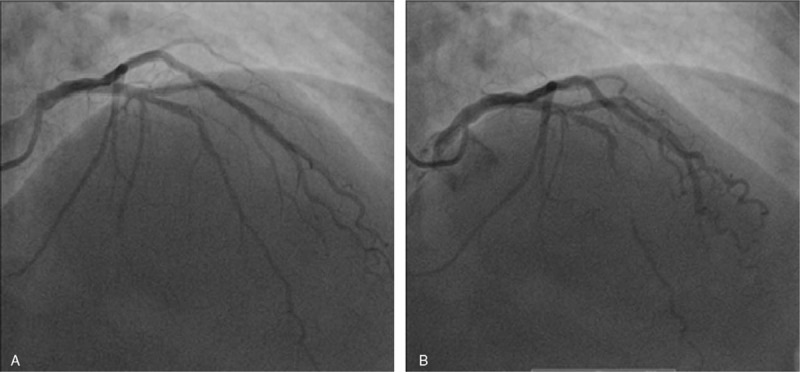
Coronary angiography, incidence right anterior oblique (RAO) during diastole (A) and systole (B).

**Figure 4:**
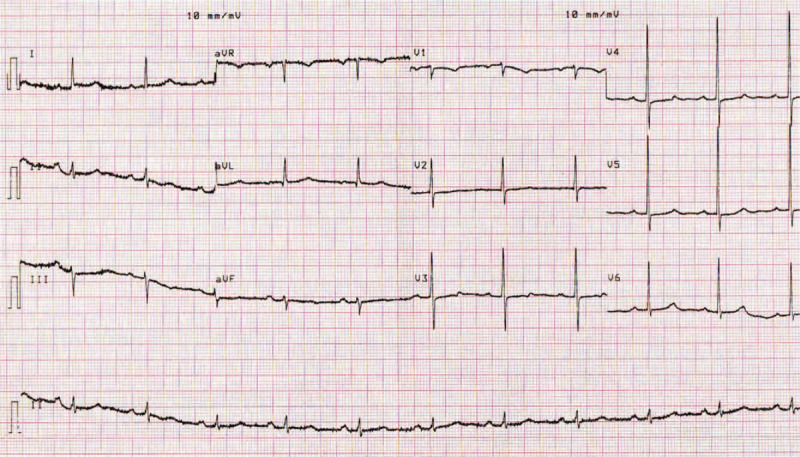
ECG taken the next day after coronary angiography showing diffuse T waves flattening. ECG = electrocardiogram.

The patient was admitted to the cardiac intensive care unit and monitored with continuous telemetry. Following her presentation, the patient was immediately started on aspirin 300 mg loading dose followed by 75 mg QD orally, clopidogrel 600 mg loading dose followed by 75 mg QD orally, atorvastatin 80 mg QD orally, and a bolus of 5000 IU of unfractioned heparin. Keeping in mind that our patient presented ECG findings compatible with type A Wellens pattern, which is highly specific (89%) for critical LAD stenosis, the patient underwent cardiac catheterization the same day. Coronary angiography showed subocclusive dynamic obstruction of the LAD due to MB (Figs. 2 and 3). The ECG taken the next day showed diffuse T waves flattening (Fig. 4).

### 2.5 Follow-up and outcome

The patient was managed conservatively. Her hospital course was uneventful and she was discharged on pharmacological therapy (clopidogrel, bisoprolol, amlodipine, atorvastatin, and metformin) with well-controlled symptoms on follow-up.

## 3 Discussion

In clinical practice it is essential to be able to read and recognize any ECG pattern suggestive of jeopardized myocardium to appropriately address and manage the situation in a timely manner. Wellens pattern has already been called a “widow-maker syndrome” ^[3]^ because it usually signals a premyocardial infarction status. Hence, Wellens sign has major diagnostic and prognostic significance.

Wellens syndrome is a clinico-electrocardiographic syndrome that was described initially by Wellens and his colleagues in the early 1980s. The incidence of the characteristic ECG pattern varies from 18% of patients with unstable angina in the original study (Wellens and de Zwaan, 1982),^[2]^ 14% in the second study by the same authors (Wellens and de Zwaan, 1989)^[4]^ to 8.8% of the patients with non-ST segment elevation myocardial infarction (Kobayashi, 2019).^[5]^

In 2002, Rhinehardt et al^[6]^ established the following criteria for differentiating Wellens syndrome from other causes of T-wave inversion in precordial leads:

- symmetric and deeply inverted T waves, in leads V2 and V3, occasionally in leads V1, V4, V5, and V6;

*or*

- biphasic T wave in leads V2 and V3;

*plus*

- isoelectric or minimally elevated (<1 mm) ST segment;
- no precordial Q waves;
- history of angina;
- pattern present in pain-free state;
- normal or slightly elevated cardiac serum markers.

There are 2 distinct morphologic subtypes of Wellens syndrome^[2,4]^:

- type A: less common; it comprises 25% of patients and is characterized by biphasic T-waves in V2, V3.
- type B: the common type; it comprises about 75% of patients and is characterized by symmetrical T-wave inversions in V2, V3, often in V4, V5, and sometimes in V6.

As originally described, Wellens pattern was associated with critical atherosclerotic stenosis of proximal LAD in 100% of the patients, with subsequent development of extensive anterior wall myocardial infarctions among 75% of patients who did not receive coronary revascularization.^[2]^ In further studies only 50% of patients had a culprit LAD lesion.^[5]^

Though atherosclerotic coronary artery disease is the major determinant of ACS, it is important to consider nonatherosclerotic causes. Our case reflects an unusual cause of myocardial ischemia—MB—as the cause of an unusual presentation of ACS—Wellens syndrome. Other miscellaneous causes for the so called pseudo-Wellens syndrome have been described, including coronary vasospasm,^[7]^ illicit drugs abuse (cocaine, marijuana),^[8]^ and stress cardiomyopathy (Takotsubo cardiomyopathy).^[9]^

In MB, a coronary artery that runs typically on the epicardium presents a transient intramyocardial course and is exposed to externally mechanical compression during ventricular systole. The overall prevalence of MB is about 19% (CI: 17%–21%); autopsy studies revealed an overall prevalence of 42% (CI: 30%–55%), computed tomography studies 22% (CI: 18%–25%), and coronary angiography 6% (CI: 5%–8%).^[10]^ Mostly asymptomatic and incidentally discovered during necroptic studies, it may cause chronic AP, ACS, coronary spasm, ventricular septal rupture, arrhythmias, exercise-induced atrioventricular conduction blocks, transient ventricular dysfunction, and sudden death.^[1]^ Moreover, the proximal portion of the bridged segment is prone to accelerated atherosclerosis, the tunneled segment being typically spared.^[11]^ Low shear stress may contribute to atherosclerotic plaque formation proximal to the bridge, whereas high shear stress may have a protective role within the tunneled segment.^[12]^

The mechanism of ischemia is complex and not fully understood. Neither nonsignificant stenosis proximal to the bridge nor systolic compression of the tunneled segment alone can sufficiently explain severe ischemia and associated symptoms.^[13]^ Alternative hypothesis and contributors are: increased diastolic/systolic flow ratio; delayed diastolic relaxation; the pressure gradient across the bridge; intramyocardial depth of the tunneled segment; the increase in sympathetic drive during stress or exercise (tachycardia leads to an increase of the systolic-diastolic time ratio at the expense of diastolic flow and increased contractility during stress further aggravates systolic and diastolic compression); endothelial dysfunction; coronary artery spasm.^[13]^

First-line management for symptomatic MB is pharmacological treatment. On the basis of the above mechanisms for ischemia, beta blockers and nondihydropyridine calcium channel blockers are preferred due to their negative inotropic and chronotropic effect. Nitrates are contraindicated because of secondary tachycardia, with reflex sympathetic stimulation.^[14]^ In cases refractory to medical management, surgical myotomy, coronary artery stenting, or coronary artery bypass grafting can be pursued. Surgical myotomy has been first reported by Binet et al in 1975^[15]^ and evolved since then, being performed nowadays with minimally invasive techniques. In bridges that take a deep subendocardial course, the right ventricle may accidentally be opened during surgery and result in right ventricle dissection.^[16]^ Coronary stenting has been first reported by Stables et al in 1995,^[17]^ but is associated with serious complications like in-stent restenosis, stent fracture, and coronary artery perforation.^[18]^

Our patient was managed conservatively on pharmacological therapy (clopidogrel, bisoprolol, amlodipine, atorvastatin, and metformin). On 3 months follow-up she reported well-controlled symptoms on medication and no further action has been taken.

The particularity of our case is the nonatherosclerotic cause of Wellens syndrome in a patient with cardiovascular risk factors (obesity, diabetes mellitus, arterial hypertension, and dyslipidemia) and no evidence of coronary artery disease. Whether nonatherosclerotic causes of Wellens syndrome carry the same ominous prognosis has yet to be addressed by further studies.

## 4 Conclusion

Timely recognition of subtle ECG findings in patients with chest pain or equivalents, such as Wellens pattern, is crucial and identifies a high-risk group of patients in whom early intervention may prevent significant morbidity and mortality. Though atherosclerotic coronary artery disease is the major determinant of acute coronary syndromes, in general, and Wellens syndrome, in particular, it is important to consider nonatherosclerotic causes. MB is an unusual cause of myocardial ischemia. Clinicians should be alert and aware when assessing patients with chest pain regarding ECG warning signs and their underlying etiology.

## Author contributions

**Conceptualization:** Anamaria Avram, Valentin Chioncel.

**Data curation:** Suzana Guberna, Irina Cuciureanu.

**Methodology:** Radu Constantin Brezeanu.

**Project administration:** Catalina Liliana Andrei.

**Software:** Anamaria Avram.

**Supervision:** Crina Sinescu.

**Writing – original draft:** Anamaria Avram.

**Writing – review & editing:** Anamaria Avram, Valentin Chioncel, Crina Sinescu.

## Abbreviations

Abbreviations: AP = angina pectoris, ECG = electrocardiogram, LAD artery = left anterior descending artery, MB = myocardial bridging.
